# The Role of Wheatgrass in Colorectal Cancer: A Review of the Current Evidence

**DOI:** 10.3390/ijms25105166

**Published:** 2024-05-09

**Authors:** Magie Tamraz, Najib Al Ghossaini, Sally Temraz

**Affiliations:** 1 Department of Nutrition and Public Health, Holy Spirit University of Kaslik, Jounieh P.O. BOX 446, Mount Lebanon, Lebanon; magietamraz@gmail.com; 2 Department of Internal Medicine, Ain Wazein Medical Village, Chouf P.O. Box 1503-210/02, Mount Lebanon, Lebanon; najibaal@hotmail.com; 3 Department of Internal Medicine, Oncology/Hematology Division, American University of Beirut Medical Center, Riad El Solh, Beirut 1107 2020, Lebanon

**Keywords:** chemotherapy, colorectal cancer, extracellular vesicles, inflammation, *Triticum aestivum*, wheatgrass

## Abstract

The etiology of colon cancer is either genetic in nature or results from inflammatory bowel diseases such as ulcerative colitis and Crohn’s disease; nevertheless, dietary habits play a crucial role in the disease. Wheatgrass is a dietary supplement that is rich in vitamins, minerals, and antioxidants which contribute to health promotion in cardiovascular diseases, liver disease, blood diseases, diabetes, and inflammatory bowel diseases, as well as in several types of cancers, such as oral squamous cell cancer, cervical cancer, and breast cancer. In colorectal cancer (CRC), the prospect that wheatgrass possesses anti-inflammatory, antioxidant, and anticancer properties, and its use as an adjunctive therapy, have been minimally investigated and evidence is still limited. In this review, we compiled the available evidence pertaining to wheatgrass and its likely impact on CRC, described the pathways of inflammation in which wheatgrass could possibly play a role, and identified future research needs on the subject.

## 1. Introduction

Colorectal cancer (CRC) is a major health concern because it ranks second in mortality among cancers worldwide [1]. The prevalence of CRC is predicted to reach up to 3.2 million new cases and 1.6 million deaths by 2040, with the majority of new cases (81%) taking place in high-income nations [1]. Heredity, genetic predisposition, food mutagens, and chronic inflammation have been implicated as possible risk factors for CRC development [2,3]. Moreover, inflammatory bowel diseases, including Crohn’s disease and ulcerative colitis, are associated with CRC development [4].

Currently, the treatment strategies for CRC include surgical resection, chemotherapy, and radiotherapy. However, these therapies are often accompanied by adverse toxic effects, chemoresistance, and off-target effects. Thus, there is an urgent need for dietary interventions that target the immune system, control inflammatory processes, and mitigate the adverse effects of chemotherapy. Nutraceuticals, which is a term derived from the words “nutrition” and “pharmaceuticals”, have been shown to exert their effects on the proliferation, metastasis, apoptosis, autophagy, and angiogenesis pathways in CRC [5]. Nutraceuticals include a wide range of dietary supplements, secondary plant metabolites, medicinal herbs, and microorganisms [6]. They control DNA-damaging factors in cancer cells, regulate DNA transcription in tumors, enhance the immune system, act as antioxidants, and possess anti-inflammatory effects [6]. For instance, curcumin, a natural polyphenol derived from the rhizome of Curcuma longa, suppresses the survival and proliferation of human colon cancer cell lines [7,8]. Resveratrol is a natural phenolic compound from the oligomeric stilbenoid group that is typically found in the human diet, such as in grapes, peanuts, berries, and grains. It exhibits anti-inflammatory, neuroprotective, antioxidant, and therapeutic effects against CRC [9]. Another example is the perennial herbaceous plant *P. quinquefolius*, sometimes known as American ginseng, which contains ginsenosides or triterpenoid saponins that prevent CRC through anti-inflammatory and apoptotic processes [10,11]. In addition, a phenolic extract of oleuropin from oleaster leaves (*Olea europaea var. Sylvestris*) induces apoptosis and inhibits cell growth in CRC cell lines [12,13]. 

Wheatgrass, also known as the sprouts of *Triticum aestivum* (*T. aestivum*), is a dietary supplement with similar nutritional potential. Recent studies, although limited, have investigated its possible anti-inflammatory and protective effects in CRC; however, a detailed description of the pathways involved is lacking. This is the first detailed review of wheatgrass that describes the possible anti-inflammatory processes targeted in CRC, its anticarcinogenic properties, and its use as an adjunctive therapy.

## 2. Methods

Data were collected from MEDLINE, PubMed, Embase, and Web of Science databases published before December 2023. The search keywords used were “colorectal cancer”, “colon cancer”, “wheatgrass”, and “*T. aestivum*”. Only articles published in English were included in this study.

## 3. Wheatgrass or Sprouts of *T. aestivum*

Wheat is a staple food that is grown worldwide. It contains carbohydrates, proteins, vitamins, minerals, and phytochemicals [14]. Through germination and sprouting, wheat grains gain nutritional value in terms of their phytochemical composition and antioxidant potential [14]. To germinate wheat grains, the grains should be subjected to moisture, a process which requires approximately 36 h to complete. Sprouts are produced 6–15 days after germination [14]. These sprouts are then cut off and juiced to provide a low-acid green juice, which has several health benefits [14]. The consumption of healthy juices, specifically those of fruit and vegetable origins, has increased with consumers’ increased awareness. Wheatgrass can be used in several forms, such as fresh juice, powder, tablets, and capsules [15]. Wheatgrass is rich in vitamins, including vitamins A and E, which are fat-soluble, and vitamins B and C, which are water-soluble, and contains minerals, mainly iron, magnesium, zinc, manganese, potassium, calcium, sodium, and copper (Table 1) [16]. 

Wheatgrass is beneficial to health because of its high chlorophyll content (chlorophyll a, 2.85 mg/g; chlorophyll b, 4.61 mg/g), xanthophylls and carotenoids content (17.31 mg/g), phenolics content, and amino acid content [14,16]. Chlorophyll found in wheatgrass is similar in structure to the hemoglobin found in human blood and has the potential to elevate levels of hemoglobin [17]. Chlorophyll derivatives, specifically chlorophyll A, B, and phytol, which are active components, possess anti-inflammatory, anti-mutagenic, and mutagen-trapping potential [15]. The anti-inflammatory properties of chlorophyll have been successfully utilized to treat several inflammation-related diseases, including dengue [18], cholangiocarcinoma [19], allergic rhinitis [20], and acne vulgaris [21]. In contrast, metallochlorophyll derivatives possess antioxidant activities that are not present in their metal-free counterparts. Carotenoids are a family of pigments that elicit protective effects through their antioxidant potential and may have a protective role in cancer by reducing growth or inducing apoptosis [22]. Two types of phenolic compounds have been identified in wheat. The free and/or soluble phenolic compounds have the potential to decrease the oxidation of low-density lipoproteins, whereas the bound/insoluble compounds have exhibited chemo-preventive potential in colon cancer [23]. Both the essential and the nonessential types of amino acids found in wheatgrass have been shown to play a role in the digestion and breaking down of food, repairing body tissues, and controlling several body functions [14]. 

Wheatgrass juice can be extracted from different wheat grains that exhibit different colors. Colored wheatgrass juices have a better nutraceutical profile than white wheatgrass juices (white < blue < purple < black). Interestingly, wheatgrass juice extracted from black wheat had the highest content of anthocyanins, chlorophyll, phenolics, proteins, essential amino acids, potassium, and antioxidants [14]. Seedlings formed by the germination of these wheat varieties are colored because of the presence of anthocyanins. Plant anthocyanins play protective roles against obesity, cancer, and cardiac diseases [24]. Wheatgrass has the potential to prevent cardiovascular and irritable bowel diseases, such as ulcerative colitis, and blood diseases, such as thalassemia, liver diseases, diabetes, and cancer, and is thus classified as a nutraceutical [25].

## 4. Mechanisms of Inflammation and Colitis-Associated CRC Development

Inflammation is the result of multiple factors such as pattern recognition receptors, the complement system, inflammasomes, antimicrobial peptides, cytokines, and chemokines. Cytokines are a diverse group of intracellular messengers with the primary role of regulating and controlling immune and inflammatory responses [26]. Cytokines that have a pro-inflammatory role include interleukin (IL)-6, IL-8, IL-1b, and tumor necrosis factor-alpha (TNF-α). Those with anti-inflammatory roles, such as IL-10, control the proinflammatory cytokine response, thereby limiting excessive inflammatory reactions. Although cytokine-mediated responses are vital in normal situations, a discrete loss of equilibrium between the formation of pro- and anti-inflammatory cytokines results in the unsuccessful resolution of the inflammatory response and chronic inflammation, which are closely related to the progression and death of patients with CRC [27]. 

Elevated levels of enzymes cyclooxygenase-2 (COX-2) and inducible nitric oxide synthase (INOS), which are associated with prostaglandin E2 (PGE2) and nitric oxide (NO), have been linked with colon carcinogenesis [28,29]. Excess NO production through INOS-induced neoplastic transformation is a vital step in carcinogenesis [30]. A significant increase in the expression of COX-2 was seen in CRC tissues of human origin but not in normal tissues. Moreover, COX-2 inhibitors block colitis-associated carcinogenesis in mice models [31]. The formation of PGE2 by COX-2 can induce proliferation and prevent apoptosis of CRC cells. Peroxisome proliferator-activated receptor δ (PPARδ) induces PGE2 through activation of PI3K-AKT signaling, thus surging the growth of adenomatous lesions in the colon. Inactivation of PPARδ has been shown to reduce colon inflammation and adenoma formation, which highlights the inflammatory role played by PPARδ [32]. PGE2 influences CXCL1, a proangiogenic chemokine that activates and recruits neutrophils, and CXCR2, the receptor of CXCL1 that allows access to myeloid-derived suppressor cells (MDSCs) [33]. MDSCs accelerate cancer growth by decreasing the activity of CD8+ T cells, highlighting the role of MDSCs in cancer immune evasion [34]. 

In addition, inflammation prompts mutation formation, with dysfunction in nuclear factor kappa B (NF-kB) pathway signaling. The NF-κB family consists of several transcription factors, including, p50, p52, p65 (RelA), c-Rel, and RelB [35]. In the cytoplasm, NF-κB forms a complex with the inhibitor IκB. As a response to certain stimuli such as cytokines and growth factors, the IκB kinase (IKK)α, IKKβ, and IKKγ complexes control IκB breakdown through the ubiquitin-proteasome system, leading to the activation and nuclear translocation of NF-κB (p50-RelA). This results in the activation of downstream gene expression that probably increases inflammation and the commencement and development of cancer [36]. Stimulation of the NF-κB pathway increases IL-6 expression in the innate immune cells inside the lamina propria. IL-6 activates signal transducers and activators of transduction-3 (STAT3). STAT3 then forms complexes with certain DNA sequences and controls the transcription of cyclin D1, a regulator of cellular proliferation; BCK-xL and survivin, regulators of survival; and vascular endothelial growth factor (VEGF), a regulator of angiogenesis [37]. Finally, IL-6/STAT3 signaling has been shown to influence immune cells infiltrating colon tumors, which are found in the tumor immune microenvironment [38]. Activation of Wnt/β-catenin has been associated with ensuing transcriptions of proliferation-associated genes such as cyclin D1 and c-Myc, which are regulated by transcription factors in the nucleus [39]. Nuclear translocation of β-catenin has been detected in >80% of CRC tumors and its subsequent activation plays a critical role in regulating cell proliferation and migration, stemness, apoptosis, autophagy, metabolism, inflammation and immunization, microenvironment, and resistance [40]. Under normal conditions, β-catenin is dissociated in the cytoplasm and its level remains the same; however, its accumulation in colon tissues induces cancer-associated colitis [41].

Sustained chronic inflammation of the large intestine is the key driver of neoplastic changes and progression that contributes to dysplasia and is considered the most critical risk factor for developing colitis-associated CRC [42]. Chronic inflammation generates oxidative stress-induced DNA damage that may activate tumor-promoting genes and inactivate tumor-suppressing genes [43]. The main gene mutation determining the progression of the colitis-associated CRC is p53 mutation, which has consequent impacts on cell cycle, DNA repair, and cell viability, as compared to APC loss of function and the WNT-beta catenin pathway activation for sporadic CRC [44]. Multiple other driver genes, such as KRAS, p53, PIK3CA, SMAD4, ARID1A, and MYC, are also involved in the following progression of sporadic CRC [45]. These genes are also involved in colitis-associated CRC, even though the timing and frequency of some of the common gene alterations are different. Since the advent of preclinical prototypes of colitis-associated CRC, various immunological messaging cascades have been identified as implicated in developing this disease, including toll-like receptors (TLRs), Janus kinase (JAK), signal transducer and activator of transcription (STAT), NF-κB, mammalian target of rapamycin complex (mTOR), autophagy, and oxidative stress [46]

## 5. Protective Roles of Wheatgrass

### 5.1. Possible Anti-Inflammatory Action of Wheatgrass

Prolonged inflammation and elevated pro-inflammatory cytokines can boost disease symptoms, such as pain, dyspnea, lethargy, cerebral impairment, and depression [47], and may induce tumor formation by promoting DNA impairment, unlimited replication, continued angiogenesis, and metastasis [48,49]. Support for the anti-inflammatory activity of wheatgrass in diseases other than cancer has been shown in a mouse model, which resulted in symptom relief of atopic dermatitis [50,51], and in one human intervention study, which showed alleviated symptoms after wheatgrass ingestion in patients with ulcerative colitis [52]. 

An ethanol extract of wheatgrass was studied in mice with colon cancer [53]. The authors evaluated the levels of mRNA of cytokines in colon tissues and found that treatment with ethanol extract of wheatgrass inhibited the expressions of TNF-α, IL-1b, IL-6, cyclin D1 and c-Myc, COX-2, INOS, and NF-κB p65 protein in the colon tissues. 

The dichloromethane extract of wheatgrass was studied in vitro to determine its anticancer effects on different cancer cell lines [54]. The authors reported that dichloromethane from wheatgrass increased cell cycle arrest and the expression of death receptors (DR-4, DR-5, and DR-6). It was also associated with increased levels of Bcl-2-associated kinase (BAX) (pro-apoptotic protein), decreased procaspase-3 and B-cell leukemia/lymphoma 2 protein (Bcl2) levels (pro-apoptotic proteins), and increased BAX/Bcl-2 ratio, indicating that its cytotoxic effects were most likely due to the stimulation of the caspase-dependent apoptotic pathway. Also, dichloromethane significantly improved the phosphorylation of extracellular signal-regulated kinase (ERK)1/2 and Jun N-terminal kinase (JNK), but not p38, and repressed the activation of NF-κB [54]. From the same group, mice treated with the dichloromethane extract of wheatgrass were also studied in vivo, and an evaluation of the serum cytokine levels of mice showed that IL-12 and levels of interferon-gamma (IFN-γ) were elevated in the treatment group [54]. IL-12 and IFN-γ are needed to constrain the development of cancer, which suggests that the immunomodulatory effect has the potential to diminish tumor growth [55].

**Figure 1:**
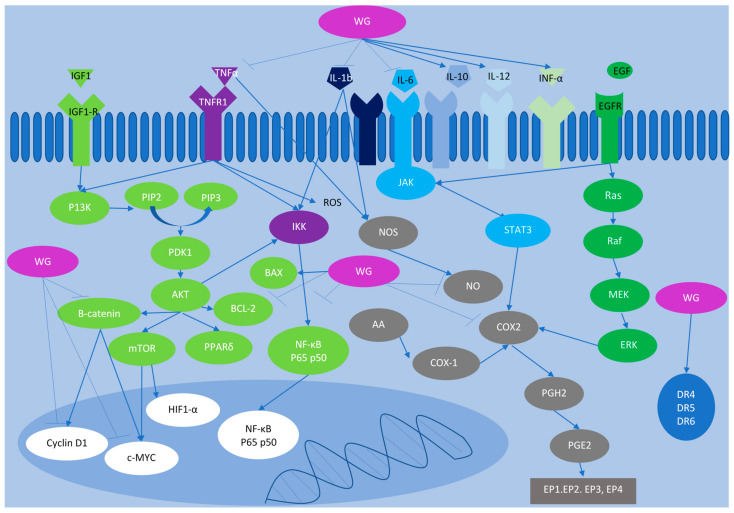
The inflammatory pathways implicated in colorectal cancer development and the role that wheatgrass plays in these pathways as an anti-inflammatory nutraceutical in colorectal cancer. Wheatgrass extracts (shown in purple) inhibit the action of numerous pro-inflammatory markers, specifically B-catenin, TNF-α, IL-6, IL-1b, NO, COX2, and NFκB, and promotes the activity of anti-inflammatory markers including DR4, DR5, DR6, IL-10, IL-12, and INF-α.WG: Wheatgrass; IGF1, insulin growth factor 1; TNFα, tumor necrosis factor alpha; EGF, endothelial growth factor; IL, interleukin; JAK, Janus kinase; STAT3, signal transducers and activators of transduction-3; PI3K, phosphatidylinositol-3 kinase; PIP, putative plasma membrane intrinsic protein subtype; PDK1, 3-phophoinositide-dependent kinase 1; AKT, protein kinase B; mTOR, mammalian target of rapamycin; PPARδ, peroxisome proliferator-activated receptor delta; IKK, IκB kinase; NF-κB, nuclear factor-κB; c-MYC, cellular myelocytomatosis oncogene product; HIF-α, hypoxia inducible factor alpha; NO, nitric oxide; NOS, nitric oxide synthase; COX, cyclooxygenase; AA, arachidonic acid; PGH2, prostaglandin H2; PGE2, prostaglandin E2; EP, prostaglandin E2 receptor; DR, death receptor; Ras, rat sarcoma virus; Raf, rapidly accelerated fibrosarcoma; MEK, mitogen-activated protein kinase; ERK, extracellular signal-regulated kinase; Bcl2, B-cell leukemia/lymphoma 2 protein; BAX, Bcl-2-associated kinase; IFN-α, interferon alfa.

In a prospective trial of 100 patients with stages II and III CRC, the outcome of daily injections of wheatgrass juice along with chemotherapy was assessed based on immune parameters and white blood cells (WBCs) [56]. Results revealed that similar mean concentrations of the cytokines IL-6, IL-8, and IL-12 were found in both study groups (those who received chemotherapy alone versus those who received chemotherapy with wheatgrass juice). However, the concentration of IL-10 significantly increased in the wheatgrass group. In addition, a significantly higher monocyte count was observed in the wheatgrass juice group, with no differences in other WBC populations between the two groups [56]. Although statistical significance was not reached, the authors reported a decrease in IL-8 concentration during wheatgrass juice injection and suggested that no significance was reached, probably because of the limited sample size and wide range of cytokine studies. The high concentration of IL-10 can be elucidated based on the increase in anti-inflammatory components such as chlorophyll, flavonoids, and superoxide dismutase in wheatgrass [57]. Moreover, it is likely that wheatgrass can diminish inflammation-related oxidative stress because of its antioxidant components [57]. Thus, the intake of wheatgrass juice could potentially reduce inflammation and oxidative stress induced by inflammation (Figure 1).

### 5.2. Possible Anticancer Properties of Wheatgrass

**Figure 2:**
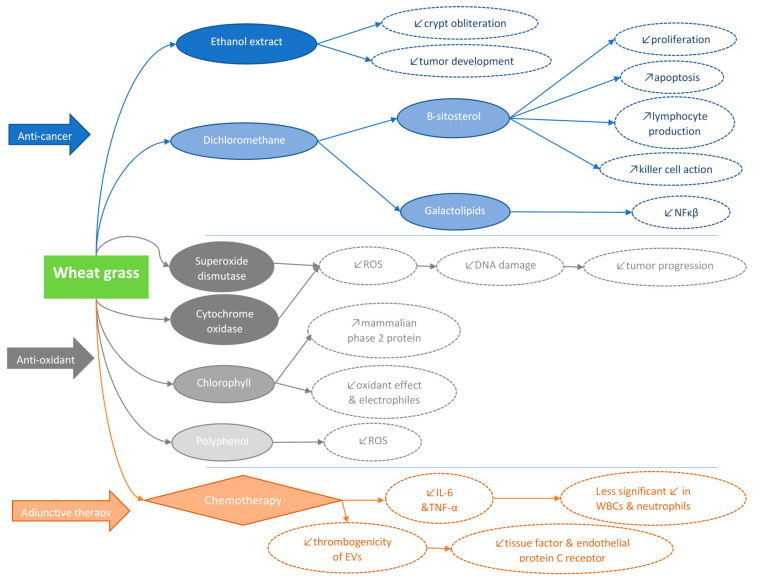
The anticancer (depicted in blue), antioxidant (depicted in grey), and adjunctive treatment properties (depicted in orange) of wheat grass. NF-κB: nuclear factor-κB; ROS: reactive oxygen species; IL-6: interleukin 6; TNF-α: tumor necrosis factor α; EVs: extracellular vesicles.

To explore the effect of wheatgrass ethanol extract on tumor development, the authors totaled the tumor counts in the colons of mice [53]. They reported that tumor counts were lower in mice treated with the ethanolic extract [53]. They also found that ethanol extracts reduced crypt obliteration and tumor development in colonic tissues [53]. The dichloromethane extract of wheatgrass reduces cell viability in a time- and dose-dependent manner in human cancer cell lines, specifically those related to the lungs, liver, gastrointestinal tract, and bones [54]. Cell viability assays and fluctuations in morphological features revealed that morphological changes are significantly associated with cancer cell death and cytotoxicity [54]. In addition, the average weight and volume of tumors in mice treated with dichloromethane were significantly lower than those in the control group [54]. Two constituents of dichloromethane extract of wheatgrass are β-sitosterol and galactolipids [58]. β-sitosterol was shown to inhibit cancer development in a renal cancer model [59] and possess an anti-proliferative as well as a pro-apoptotic potential through induction of cell cycle arrest and cell demise [60]. Moreover, β-sitosterol and β-sitosterol glycoside complexes derived from plant origins act by targeting certain T-helper lymphocytes and augmenting lymphocyte production and natural killer cell action [61]. In contrast, galactolipids inhibit cytoplasmic NF-κB activity and have anticancer potential [62]. These studies suggest that β-sitosterol and galactolipids constituents of dichloromethane may be primarily accountable for the anticancer action of wheatgrass (Figure 2). 

Wheatgrass has also shown anticancer activity against other types of cancer. In oral squamous cell carcinoma, 41.4% cell inhibition was seen at a dosage of 1000 µg/mL of wheatgrass extract in 24 h [63]. When HeLa cells derived from cervical cancer were combined with the methanolic extract obtained from the leaves of *T. aestivum*, a cytotoxic effect was observed: an amount of 19.5–10,000 μg/mL resulted in an increased percentage of inhibition from 11.9–72.3% [64]. In a prospective matched control trial involving patients with breast cancer, wheatgrass juice, in addition to chemotherapy, showed myelotoxicity reduction potential and decreased the dose and requirement for granulocyte colony-stimulating factor support without affecting the results of chemotherapy [65]. Abscisic acid is also a component of wheatgrass that has the potential to neutralize the effects of chronic gonadotropins and other similar compounds produced by cancer cells [66]. The alkaline pH of wheatgrass influences its anticancer properties by reducing the number of microbes in the diet, which decreases the occurrence of secondary infections, and cancer cells succumb to the highly alkaline milieu [66].

### 5.3. Possible Antioxidant Activity of Wheatgrass

Intracellular reactive oxygen species (ROS) correlate with cell proliferation arrest. In addition, oxidative stress resulting from external stimuli has been associated with the initiation of transcription factors and induction of apoptosis. Free radicals cause changes in DNA sequences, such as mutations, deletions, gene amplifications, and rearrangements, which may be responsible for initiating apoptosis, leading to cell death, activating proto-oncogenes, or inactivating tumor suppressor genes [67]. Wheatgrass could impede the production of ROS and prevent oxidative DNA damage, thereby preventing tumor progression (Figure 2). Wheatgrass contains enzymes with antioxidant properties, such as superoxide dismutase and cytochrome oxidase, which have the potential to convert free radicals, such as ROS, into hydrogen peroxide and oxygen molecules [68]. Chlorophyll, the major component of wheatgrass, can stimulate mammalian phase 2 proteins, which in turn protect cells from the damaging effects of oxidants and electrophiles [69]. Methyl phosphorbide, isolated from the ethanol extract of wheatgrass, has shown both antioxidant and cytotoxic effects on HeLa and Hep G2 cells. Cancer cell survival decreases with increasing concentrations of methylphorbide [70]. In addition, polyphenols in wheatgrass reduce the effects of ROS and may possibly limit the likelihood of cancerous diseases [71].

### 5.4. Wheatgrass as Adjunctive Therapy in CRC

Therapy for early-stage disease entails resection of the tumor and regional lymph nodes. The 5-year disease-free survival rate of early-stage CRC is 95% [72]. For advanced stages, the disease-free survival rate decreases from 90% to 50% for surgery alone, necessitating the use of chemotherapy, where only approximately 17–20% remain alive [73]. Chemotherapy entails the use of both single-agent therapy, consisting of fluoropyrimidine (5-FU), and multiple-agent therapy, encompassing one or more drugs, including oxaliplatin, irinotecan, and capecitabine. Chemotherapy may induce and enhance inflammation [74], decrease immunity, induce vascular injury, and increase risk [75], thus resulting in negative repercussions in patients with CRC. Wheatgrass consumption may provide synergistic benefits in CRC therapy and reduce the side effects of chemotherapy in patients with CRC [76]. 

Chemotherapy-related leukopenia and neutropenia are major dose-limiting toxicities associated with chemotherapy. Severe neutropenia (<0.5 K/uL) is a major contributor factor for infections [77]. Thus, therapies that support the immune system, particularly the WBCs, are important during chemotherapy. Wheatgrass may possess this potential, as demonstrated in two previous studies. The authors suggested a converse relationship between pro-inflammatory cytokines and WBC counts during chemotherapy. The levels of cytokine IL-6 were increased in patients with chemotherapy-induced myelosuppression [78]. Similarly, IL-6 and TNF-α were shown to be elevated almost simultaneously, with a decrease in neutrophil numbers after chemotherapy administration [79]. Wheatgrass juice ingestion revealed that the alteration in WBC counts and, specifically, the changes in neutrophil counts from baseline throughout chemotherapy treatment differed between patients who were administered wheatgrass juice and those who were not [56]. Thus, although WBC counts decreased in both groups during treatment, the decrease in WBC and neutrophil counts was less significant in the group receiving wheatgrass. 

Extracellular vesicles (EVs), including exosomes and microvesicles, are involved in intercellular communication with the tumor microenvironment (TME), thus leading to the differentiation, proliferation, migration, and invasion of cells, as well as tumor progression [80]. In colon cancer, EVs are rich in specific proteins [81] and cell cycle-related mRNAs that enhance endothelial cell proliferation [82]. EVs have been shown to moderate the immune activity of macrophages, thus reducing their tumor-restrictive capacity [83]. Moreover, EVs obtained from patients with stage II–IV colon cancer have more thrombogenic properties than those obtained from healthy controls [84]. Thus, these EVs may mirror the body’s response to distress and explain the impact of nutritional support during chemotherapy. In a recent study, the authors explained the effects of chemotherapy with or without wheatgrass juice administration on the features of EVs of patients with CRC [85]. Their results revealed that elevated numbers of endothelial EVs, which may be a sign of vascular endothelial cell impairment, were present in patients with chemotherapy-treated CRC. In addition, the thrombogenicity of EVs decreased in patients receiving wheatgrass juice, and the levels of tissue factor and endothelial protein C receptor were significantly decreased (Figure 2). Angiogenesis, which is controlled by vascular endothelial growth factor receptors (VEGFR), plays a major role in tumor proliferation and metastasis, thereby enhancing the survival and growth of CRC [86]. The levels of VEGFR-1 and a high number of growth factors/pro-inflammatory cytokines were increased in the EVs of patients receiving chemotherapy alone [85]. 

## 6. Other Forms of Wheat with Anticancer and Anti-Inflammatory Properties

The protective properties of wheat are not limited to its wheatgrass form. Other forms of wheat such as fermented wheat and wheat aleurone have been shown to have beneficial effects on patients with CRC. In vitro studies of fermented wheat germ extract have shown that this form of wheat possesses anticancer potential by stimulating apoptosis and inhibiting proliferation and angiogenesis [87]. Moreover, supplementation with wheat germ extract for 6 months resulted in significantly fewer progression-related events. Specifically, new recurrences (l3.0 vs. 17.3%, *p* < 0.01), new metastases (7.6 vs. 23.1%, *p* < 0.01), and deaths (12.1 vs. 31.7%, *p* < 0.01) were significantly lower in patients receiving wheat germ extract as compared to controls. Progression-free survival and overall survival were significantly better in the group treated with fermented wheat germ extract [88]. Wheat aleurone is the sole cell layer in the internal spot of the bran and encompasses the majority of minerals, vitamins, phenolic antioxidants, and lignans of the wheat grain. Fermented wheat aleurone induces apoptosis and blocks the cell cycle in HT29 colon cells [89]. Wheat aleurone acts as a secondary chemoprotective factor by regulating cell growth and survival [90]. Moreover, fermented aleurone acts as a principal preventive agent by stimulating mRNA expression and enhancing the activity of enzymes involved in the removal of carcinogens and antioxidative defense [91]. 

## 7. The Countereffects of Wheat

Coeliac disease is an autoimmune disease that affects the small intestine of genetically prone individuals and is exacerbated by the ingestion of gluten, a protein found mainly in wheat, rye, and barley. Coeliac disease affects approximately 1% of the world’s population [92]. Its harmful effects are attributed to intestinal villus atrophy, which results in malnutrition and malabsorption of essential nutrients [92]. A gluten-free diet that limits the consumption of gluten from wheat and other sources is a treatment strategy for coeliac disease. Duodenal inflammation is imminent upon reintroduction of gluten; therefore, these patients should follow a long-term gluten-free diet. Irritable bowel syndrome is a functional bowel disease characterized by abdominal pain, diarrhea, bloating, and constipation and affects approximately 5% of the world’s population [92]. Gluten from wheat has also been implicated in irritable bowel syndrome, with approximately 30% of patients generating symptoms upon gluten consumption [93]. Thus, gluten sensitivity should be assessed among patients with CRC prior to the ingestion of wheatgrass, which contains gluten. 

## 8. Conclusions and Future Perspectives

The current evidence suggests that the antioxidants in wheatgrass may have anti-inflammatory and protective effects in CRC, and a diet rich in fruits and vegetables, including wheatgrass, is generally associated with lower cancer risk. However, direct evidence linking wheatgrass consumption to the prevention of CRC is still limited. Although the popularity of wheatgrass continues to increase and the benefits seen in small trials need to be proven in larger, randomized population-based trials before clinical recommendations are provided to the public, the fact that wheat is a staple food that is widely available and can be grown and domesticated worldwide is a positive feature of the crop. Moreover, obtaining grass from wheat can be fulfilled by most people. Thus, if supported by clinical trials, wheatgrass juice supplementation could become a basic component of the healthy diet of individuals at risk of developing CRC, patients diagnosed with CRC, and healthy individuals as a preventive measure. 

Several topics related to CRC are currently being investigated. The gut microbiome is one such topic, and research has shown its involvement in CRC. Thus, further studies on the role of wheatgrass and its involvement in the gut microbiome of patients with CRC should be conducted. Another topic pertains to the role of immunotherapy in the treatment of CRC alongside chemotherapy. Preliminary studies have shown that patients with compromised immune systems are more likely to benefit. Thus, wheatgrass juice supplementation may be protective, particularly in patients with inflammation- and immune-related CRC and antioxidant-related CRC. However, this requires validation in large clinical trials that select patients with CRC with compromised immune, inflammatory, and antioxidant systems.

## Figures and Tables

**Table 1:** Vitamin, mineral, and chemical composition of wheatgrass.

Components of Wheatgrass	Amount Found in Wheatgrass	Role
Vitamins		
Vitamin C	9.3 mg/100 g	Anti-oxidative Scavenging for free radicals
Vitamin B3	6.71 mg/100 g	Anti-oxidative Scavenging for free radicals
Vitamin B6	6.45 mg/100 g	Anti-oxidative Scavenging for free radicals
Vitamin B1	5.79 mg/100 g	Anti-oxidative Scavenging for free radicals
Vitamin B2	5.73 mg/100 g	Anti-oxidative Scavenging for free radicals
Vitamin B10	5.12 mg/100 g	Anti-oxidative Scavenging for free radicals
Vitamin B4	4.15 mg/100 g	Anti-oxidative Scavenging for free radicals
Minerals		
Iron	66.83 ppm	Important role in electron transport chain Cofactor in various enzymes such as peroxidases, cytochromes, and xanthine oxidases
Magnesium	64.107 ppm	Prominent constituent of chlorophyll; present as central element in porphyrin ring
Zinc	32.93 ppm	Scavenging free radicals Activation of various enzymatic functioning of plants Triggering heavy metal-induced protein and lipid oxidation
Manganese	26.89 ppm	Scavenging for free radicals
Potassium	25.541 ppm	Transmission of nerve signals, and plays a role in muscle contraction, fluid balance, and various chemical reactions
Calcium	17.238 ppm	Cofactor in enzymatic reaction such as oxidation of fatty acids and maintenance of mineral homeostasis
Sodium	5.012 ppm	Maintains normal fluid levels outside cells
Copper	4.3 ppm	Acts as an antioxidant Reduces free radicals
Aluminum	1.231 ppm	Induces root growth and enhances enzymatic functions and nutrient intake in plants
Selenium	1.101 ppm	Component of antioxidant enzymes such as catalase, glutathione peroxidase, and superoxide dismutase
Chromium	0.101 ppm	Plays a role in glucose and lipid metabolization
Cobalt	0.013 ppm	Primary constituent of vitamin B12-induced erythropoietin and metabolizing methionine
Chemical composition		
Carbohydrates	361 mg/g	
Total sugars	17.75 mg/g	
Reducing sugars	13 mg/g	
Ash content	14%	
Moisture content	3.5%	
Crude fats	5.45%	
Crude protein	21.87%	
Crude fibers	1.4%	

## Data Availability

Not applicable.
